# Environmental Cancer Risk Scenarios for Lebanon: Quantitative Sensitivity Analysis and a Qualitative Genetic Susceptibility Framework

**DOI:** 10.3390/toxics14100921

**Published:** 2026-10-17

**Authors:** Magie Tamraz, Rihab Nasr, Sally Temraz

**Affiliations:** 1 Department of Epidemiology and Population Health, Faculty of Health Sciences, American University of Beirut, Riad El Solh, Beirut 1107, Lebanon; mnt04@mail.aub.edu; 2 Department of Anatomy, Cell Biology and Physiological Sciences, Faculty of Medicine, American University of Beirut, Riad El Solh, Beirut 1107, Lebanon; 3 Division of Oncology/Hematology, Department of Internal Medicine, American University of Beirut Medical Center, Riad El Solh, Beirut 1107, Lebanon

**Keywords:** cancer risk, conflict-related environmental contamination, environmental exposure modeling, environmental risk assessment, genetic susceptibility, Lebanon, Monte Carlo simulation, nitrate contamination, perchlorate exposure, scenario-based modeling

## Abstract

Background: Environmental exposures and biological susceptibility contribute to the complexity in cancer vulnerability, particularly in settings where comprehensive environmental monitoring and longitudinal exposure data are limited. Scenario-based modeling can provide a structured approach for exploring potential environmental cancer risks under uncertainty and identifying priorities for future research and surveillance. Methods: We developed a scenario-based environmental cancer risk framework for Lebanon incorporating three exposure domains—perchlorate contamination, nitrate/nitrite exposure, and conflict-derived environmental contamination— and a qualitative Genetic Susceptibility Index (GSI). Monte Carlo simulations with 100,000 iterations were used to propagate uncertainty in literature-derived epidemiological effect estimates for colorectal and lung cancers under alternative hypothetical scaling assumptions. The GSI was used to organize qualitative susceptibility pathways but was not incorporated as a numerical modifier in the simulations. Thyroid cancer was evaluated separately through an illustrative hypothetical sensitivity analysis using author-defined relative-effect ranges because direct epidemiological estimates linking perchlorate exposure to thyroid cancer incidence were unavailable. Results: For colorectal cancer, the mean simulated relative effect increased from 3.79% (95% UI: 1.90–5.70%) under the low-exposure-contrast scenario to 7.72% (95% UI: 3.83–11.71%) under the moderate scenario and 16.09% (95% UI: 7.81–24.80%) under the high scenario, which applied the full published log-relative effect. For lung cancer, corresponding mean simulated relative effects were 2.18% (95% UI: 1.01–3.36%), 4.41% (95% UI: 2.02–6.83%), and 9.03% (95% UI: 4.09–14.13%). Under the full published exposure contrast, 92.34% of colorectal cancer simulations exceeded a 10% relative-effect threshold and 94.52% of lung cancer simulations exceeded a 5% threshold. The thyroid cancer analysis produced mean simulated relative effects of 1.03%, 3.50%, and 7.51% under the low-, moderate-, and high-scenario assumptions, respectively; these outputs were conditional on the prespecified hypothetical ranges. Conclusions: This framework demonstrates how alternative exposure-contrast assumptions can influence the distribution of literature-derived relative effects in a data-limited setting. By combining quantitative exposure-contrast sensitivity analysis with a qualitative genetic susceptibility framework, the approach may support environmental health planning, surveillance prioritization, and the design of future population-based studies in Lebanon. The thyroid cancer results illustrate model behavior under author-defined hypothetical assumptions and do not constitute epidemiological estimates of perchlorate-associated thyroid cancer risk. The findings are exploratory and should not be interpreted as estimates of cancer incidence, population-attributable risk, or causal effects in the Lebanese population.

## 1. Introduction

Environmental contamination is increasingly recognized as a major contributor to global disease burden, particularly in regions affected by industrial accidents, armed conflict, and inadequate environmental governance. Exposure to hazardous chemicals can have long-term consequences on human health, including increased risks of endocrine disorders, respiratory diseases, reproductive dysfunction, and cancer [1]. Contemporary environmental health research has increasingly shifted from evaluating individual toxicants in isolation toward understanding cumulative exposures and the biological factors that modify individual susceptibility to their effects. This perspective is reflected in the emerging exposome framework, which emphasizes the lifelong interaction between environmental exposures and host biological characteristics in shaping disease risk [2,3].

Lebanon has experienced longstanding environmental challenges characterized by multiple and overlapping sources of pollution, including inadequate waste management, deteriorating air quality associated with traffic and diesel generator emissions, contamination of water resources, and the persistence of agricultural and industrial pollutants in environmental media [4,5,6]. These long-term environmental problems have been compounded by recent disasters and conflict-related events. The Beirut Port explosion on 4 August 2020, involving approximately 2750 tons of ammonium nitrate, was one of the largest non-nuclear explosions in history and resulted in widespread environmental disruption, generating concerns regarding air quality deterioration and long-term public health consequences [7,8]. In parallel, ongoing military activity in southern Lebanon has raised concerns regarding additional conflict-related environmental contamination, including exposure to incendiary agents such as white phosphorus [9].

Within this complex environmental landscape, three exposure domains were selected for the present modeling framework: perchlorate contamination, nitrate/nitrite exposure, and conflict-derived environmental contamination. These domains were specifically selected to explore the potential health implications of environmental exposures that are newly recognized or of emerging concern in the Lebanese context, for which population-level exposure and long-term health consequences remain incompletely characterized in Lebanon. Other environmental contaminants, including heavy metals and diesel-generator emissions, may also contribute to environmental health risks in Lebanon but were beyond the specific scope of the present study. The selected exposure domains differ in their sources, environmental persistence, and biological effects, yet may converge on shared molecular pathways implicated in carcinogenesis, including oxidative stress, chronic inflammation, endocrine disruption, and genomic instability. These shared pathways provide a conceptual basis for organizing the exposure domains; they do not establish that each selected contaminant has a demonstrated causal association with the cancer endpoints considered.

Perchlorate is a highly soluble environmental contaminant associated with fertilizers, explosives, rocket propellants, and other anthropogenic sources. Its persistence and mobility in water systems can facilitate contamination of groundwater and surface water and subsequent entry into the food chain [10]. Although direct environmental measurements of perchlorate in Lebanon remain limited, its potential relevance in settings affected by military activity, explosive residues, and agricultural chemical use warrants consideration as a candidate exposure domain [11,12]. Perchlorate competitively inhibits the sodium–iodide symporter in the thyroid gland, reducing iodide uptake and potentially disrupting thyroid hormone synthesis [13]. While perchlorate is not an established human carcinogen, its effects on thyroid physiology support further investigation of potential health consequences without establishing a causal relationship with thyroid cancer [14]. Accordingly, perchlorate was included in the present framework as a hypothetical exposure domain with a separately evaluated thyroid cancer sensitivity analysis, rather than as an established carcinogenic exposure or a quantitatively parameterized epidemiological association.

Nitrate contamination of groundwater and surface water arises primarily from agricultural fertilizer application, wastewater discharge, livestock production, and industrial activities, resulting in increased human exposure through drinking water and the food chain [15]. In Lebanon, concerns regarding environmental contamination have been heightened by recent industrial disasters, including the Beirut Port explosion, which underscored the potential public health implications of large-scale chemical releases [7]. However, the extent to which the explosion contributed to sustained population-level nitrate exposure remains uncertain, and the present analysis does not quantify such an effect. Following ingestion, nitrate can undergo endogenous conversion to nitrite and subsequently to N-nitroso compounds, a class of chemicals with recognized carcinogenic potential in experimental models [7,16]. Epidemiological studies have reported associations between long-term nitrate exposure and increased risks of certain gastrointestinal malignancies, particularly colorectal cancer, although findings remain heterogeneous across populations and exposure settings [16]. For the present quantitative analysis, the colorectal cancer parameter was taken from a published study comparing the highest and lowest categories of long-term drinking-water nitrate exposure; the model did not estimate nitrate exposure levels or exposure distributions in Lebanon.

Conflict-derived environmental contamination represents a third and less well-characterized exposure domain. Military activities generate complex mixtures of environmental pollutants, including particulate matter, combustion by-products, heavy metals, explosive residues, and incendiary-agent derivatives, which may persist in environmental media following armed conflict [17]. White phosphorus has received particular attention in southern Lebanon as one example of conflict-associated contamination because of concerns regarding its environmental deposition following military use [9]. Although white phosphorus itself has not been established as a human carcinogen, its presence may serve as a marker of broader conflict-associated environmental pollution rather than an isolated toxicological exposure. Within this broader conceptual exposure domain, particulate matter and combustion-derived pollutants provide a more established epidemiological basis for quantitative cancer-risk parameterization; accordingly, PM_2.5_ was used as the quantitatively parameterized component for the lung cancer analysis. The published PM_2.5_ association was used as a literature-derived parameter under hypothetical exposure-contrast assumptions and was not interpreted as an estimate of exposure to, or cancer risk from, white phosphorus or conflict-derived pollution specifically in Lebanon. Chronic exposure to combustion-derived pollutants and fine particulate matter has been linked to biological processes implicated in carcinogenesis, including oxidative stress, persistent inflammation, DNA damage, and dysregulation of cellular repair pathways [18,19]. These mechanisms are particularly relevant for respiratory malignancies, including lung cancer.

Importantly, cancer risk is not determined solely by the magnitude of environmental exposure. Substantial inter-individual variation exists in biological responses to environmental toxicants, reflecting differences in genetic susceptibility. Genetic susceptibility frameworks propose that inherited variation in biological pathways involved in xenobiotic metabolism, DNA repair, oxidative stress regulation, and endocrine function may contribute to differences in biological responses to environmental exposures [20,21]. Variants in genes involved in xenobiotic, DNA repair, and thyroid physiology have been implicated as potential modifiers of environmentally mediated carcinogenesis [22]. In the present framework, these susceptibility pathways were used to define qualitative GSI strata rather than quantitative genetic effect modifiers, reflecting the absence of sufficiently robust genotype-specific interaction estimates for the selected exposure–cancer relationships. The GSI therefore serves as a qualitative organizing framework and does not quantify individual genetic risk or modify the numerical outputs of the Monte Carlo simulations.

Despite increasing recognition of the importance of genetic susceptibility in modifying responses to environmental exposures, significant knowledge gaps remain. Existing environmental health studies have largely focused on individual contaminants or isolated environmental events, often without considering cumulative exposure patterns or variability in host susceptibility. This limitation is particularly relevant in Lebanon, where populations may simultaneously experience chronic environmental pollution, agricultural contamination, industrial accidents, and conflict-related exposures. The absence of long-term prospective exposure cohorts further complicates efforts to directly quantify population-level cancer risks attributable to environmental contamination.

In settings where long-term prospective cohorts, comprehensive environmental monitoring, and biomonitoring data are limited, scenario-based modeling approaches provide a transparent framework for exploring potential environmental health implications under uncertainty [23,24]. Rather than predicting future cancer incidence, these models can be used to examine alternative environmental exposure conditions and qualitatively defined susceptibility profiles, while propagating uncertainty in available epidemiological effect estimates. The interpretation of such analyses depends on clearly distinguishing uncertainty propagated from published epidemiological estimates from outcomes generated using hypothetical, author-defined assumptions.

This study therefore presents a scenario-based environmental cancer risk framework that considers environmental exposure scenarios and qualitative genetic susceptibility profiles as conceptual dimensions, while using probabilistic simulation to propagate uncertainty in selected literature-derived cancer risk estimates. The framework integrates perchlorate contamination, nitrate/nitrite exposure, and conflict-derived environmental contamination with qualitatively defined genetic susceptibility strata. For colorectal and lung cancer, Monte Carlo simulations were used to evaluate how alternative hypothetical scaling factors applied to published log-relative effect estimates influence the distribution of simulated relative effects. These analyses do not incorporate Lebanese-specific exposure distributions or quantitatively model genetic susceptibility. Thyroid cancer was evaluated separately through an illustrative hypothetical sensitivity analysis using author-defined relative-effect ranges, because direct epidemiological estimates linking perchlorate exposure with thyroid cancer incidence were unavailable. The thyroid ranges were not derived from epidemiological effect estimates, and the mechanistic evidence provides biological context rather than quantitative validation of those ranges. Breast cancer and other cancer types were not modeled because the selected exposure domains were not parameterized for these endpoints within the present framework. The framework is intended as an exploratory approach for organizing environmental cancer risk hypotheses, evaluating sensitivity to alternative exposure assumptions, and identifying priorities for environmental monitoring, surveillance, and future population-based research in Lebanon. The findings should be interpreted as assumption-dependent sensitivity-analysis results rather than estimates of actual cancer risk or future cancer burden in the Lebanese population.

## 2. Materials and Methods

### 2.1. Study Design

This study employed a Monte Carlo simulation framework to explore uncertainty in selected environmental cancer risk estimates under alternative exposure scenarios. The modeling approach combined a qualitative genetic susceptibility framework with a quantitative sensitivity analysis of alternative exposure-contrast assumptions. The analysis focused on thyroid, colorectal, and lung cancers because of their mechanistic relevance to perchlorate contamination, nitrate/nitrite exposure, and conflict-derived environmental contamination, respectively [13,16,18]. The thyroid cancer component was treated separately as an illustrative hypothetical sensitivity analysis because robust epidemiological estimates linking perchlorate exposure to thyroid cancer incidence were unavailable for quantitative parameterization.

The quantitative component did not constitute a calendar-year-specific projection of cancer incidence or population cancer burden. Instead, published epidemiological effect estimates were used as literature-derived parameters for probabilistic uncertainty propagation under alternative exposure-contrast assumptions for colorectal and lung cancer. GLOBOCAN 2022 estimates were not used as model inputs for the quantitative analysis and are therefore not included in the simulation framework [25].

The objective of the quantitative analysis was not to predict future cancer incidence or estimate absolute cancer burden in Lebanon, but rather to characterize uncertainty around selected literature-derived relative risk estimates and to evaluate the sensitivity of these estimates to alternative assumptions regarding the magnitude of the relevant exposure contrast. Accordingly, the simulated relative changes represent uncertainty propagated from published effect estimates under specified mathematical assumptions; they are not estimates of actual exposure-related risk in the Lebanese population, effects of specific exposure reductions, or predictions of future cancer incidence.

### 2.2. Environmental Exposure Framework

Environmental exposures were represented using a scenario-based Environmental Exposure Index (EEI) developed to capture uncertainty across three exposure domains relevant to the Lebanese environmental context:

1. Perchlorate contamination;
2. Nitrate/nitrite exposure;
3. Conflict-derived environmental contamination.

These exposure domains were selected based on evidence of environmental occurrence, biological plausibility, and potential relevance to carcinogenic pathways identified in previous epidemiological and toxicological studies. Perchlorate contamination was included because of its established effects on thyroid physiology through inhibition of the sodium–iodide symporter. Nitrate and nitrite exposure were included because of their role in endogenous N-nitroso compound formation and epidemiological evidence linking nitrate exposure to colorectal cancer risk. Conflict-derived environmental contamination was included as a composite exposure domain representing particulate matter, combustion products, explosive residues, incendiary-agent derivatives, and other pollutants generated during military activities and armed conflict. Within this broader domain, particulate matter was considered the component for which a quantitative epidemiological cancer-risk estimate was available for parameterization of the lung cancer endpoint.

Because comprehensive nationwide environmental monitoring data remain limited, the EEI was modeled as a scenario-based construct rather than a direct measure of population exposure. Three environmental scenarios were evaluated:

- Low exposure (hypothetical mitigation scenario);
- Moderate exposure (intermediate hypothetical scenario);
- High exposure (persistent contamination scenario).

For the quantitative colorectal and lung cancer sensitivity analyses, the qualitative EEI categories were mapped to hypothetical scaling factors of 25%, 50%, and 100% of the corresponding log-relative effect. For the lung cancer analysis, they represent hypothetical fractions of the published effect associated with a 10 μg/m^3^ increase in PM_2.5_. Under the assumed log-linear exposure–response relationship, these scenarios therefore corresponded to 2.5, 5, and 10 μg/m^3^, respectively. For the colorectal cancer analysis, the published exposure contrast represented the comparison between the highest and lowest levels of drinking-water nitrate exposure. Because this estimate is categorical rather than a per-unit concentration coefficient, the 25%, 50%, and 100% values were applied as hypothetical scaling factors to the published log-hazard ratio. They should not be interpreted as defined fractions of the nitrate concentration difference or estimates of the effects of specified nitrate reductions. Thus, the scaling factors for colorectal cancer represent hypothetical mathematical scaling of the published log-hazard ratio, whereas those for lung cancer correspond to hypothetical PM_2.5_ contrasts under the assumed log-linear exposure–response relationship. Thyroid cancer was evaluated separately using author-defined hypothetical effect ranges, rather than a literature-derived epidemiological effect estimate. These ranges were selected solely to illustrate the sensitivity of model outputs to alternative assumed effect magnitudes and were not intended to represent empirically established thyroid cancer effects.

No Lebanese population exposure concentrations, exposure prevalence estimates, or annual exposure trajectories were assigned to the EEI categories. Accordingly, the EEI should not be interpreted as a measured environmental exposure index or as a population-level exposure distribution.

### 2.3. Genetic Susceptibility Framework

Genetic susceptibility was incorporated through a Genetic Susceptibility Index (GSI), developed as a qualitative population-level framework representing biologically plausible variation in responses to environmental exposures. Because nationally representative genotype prevalence data and population-specific exposure–susceptibility estimates are unavailable in Lebanon, candidate genes were identified from published international epidemiological and mechanistic literature on genetic susceptibility and environmentally mediated carcinogenesis (Table 1). These studies were used to establish biological plausibility and pathway relevance rather than to infer Lebanese-specific genetic effects.

The GSI included six candidate genes representing biological pathways relevant to the selected exposure domains, including xenobiotic metabolism, nitrosamine metabolism, DNA repair, metabolism of combustion-derived carcinogens, and thyroid iodide transport (Table 1). The genes were selected as representative pathway markers based on published evidence of their potential relevance to environmentally mediated carcinogenic processes and were not intended to constitute a comprehensive or validated genetic susceptibility panel. Although oxidative stress is an important pathway in environmentally mediated carcinogenesis, antioxidant-response genes were not included as separate candidate loci because the GSI was designed as a focused, exploratory framework rather than a comprehensive assessment of genetic susceptibility. The candidate genes were therefore used to establish biologically relevant susceptibility pathways rather than to calculate a composite genetic risk score or numerical susceptibility multiplier.

Three qualitative susceptibility strata were evaluated:

- Low susceptibility;
- Intermediate susceptibility;
- High susceptibility.

These strata were used as qualitative scenario-defining constructs for comparative analyses rather than as measures of genotype prevalence or quantitative genetic risk. No numerical effect modifiers or genotype-specific risk estimates were assigned to the GSI, which served solely as a conceptual framework for representing biologically plausible variation in population susceptibility under alternative environmental exposure scenarios. Accordingly, the GSI categories were not used to mathematically modify the literature-derived relative risk or hazard ratio distributions in the Monte Carlo simulations.

The nine combinations (Table 2) represent qualitative exposure–susceptibility scenarios used to structure comparative interpretation within the framework. The GSI remained qualitative and was not assigned numerical genetic effect modifiers. For the colorectal and lung cancer endpoints, the EEI categories were linked to hypothetical scaling factors of 25%, 50%, and 100% applied to the published log-relative effects for sensitivity analysis; however, these quantitative assumptions were not stratified by GSI and did not generate nine independently parameterized Monte Carlo simulations. Thus, the nine EEI × GSI combinations define the conceptual scenario space, whereas the quantitative analysis evaluates exposure-contrast sensitivity independently of genetic-stratum-specific effect modification.

### 2.4. Cancer-Specific Risk Parameterization

Cancer-specific epidemiological effect estimates were derived preferentially from published systematic reviews, meta-analyses, and large population-based cohort studies. Relative risk distributions were parameterized using reported effect estimates and corresponding 95% confidence intervals (Table 3). For each parameter, the exposure contrast reported in the source study was retained as the basis for interpretation. The colorectal cancer estimate from Schullehner et al. [16] represents the comparison between the highest and lowest levels of drinking-water nitrate exposure, while the lung cancer estimate from Hamra et al. [32] represents the relative risk associated with a 10 μg/m^3^ increase in ambient PM_2.5_. These estimates were therefore treated as literature-derived reference parameters for uncertainty propagation rather than as Lebanon-specific exposure–response functions. The published effect estimates were not statistically transported, recalibrated, or otherwise adjusted to the Lebanese population; they were used as literature-derived parameters for exploratory sensitivity analysis under alternative exposure-contrast assumptions.

### 2.5. Monte Carlo Simulation

Monte Carlo simulations were performed using 100,000 iterations for each quantitative exposure-contrast scenario, a number selected to ensure stable estimation of summary statistics and uncertainty intervals while minimizing Monte Carlo sampling error.

For each cancer endpoint:

1. A relative risk or hazard ratio was sampled from the corresponding log-normal distribution parameterized from the published point estimate and 95% confidence interval.
2. The sampled log-relative effect was scaled according to the assumed scenario factor: 25% (low), 50% (moderate), or 100% (high) of the published log-relative effect. This scaling was applied on the logarithmic relative-risk scale. For colorectal cancer, these values represent hypothetical scaling factors applied to the published log-hazard ratio, not defined fractions of the underlying nitrate concentration contrast. For lung cancer, they represent hypothetical fractions of the published effect associated with a 10 μg/m^3^ increase in ambient PM_2.5_, under the assumed log-linear exposure–response relationship.
3. The scaled log-relative effect was exponentiated to obtain the scenario-specific relative effect, which was then converted to a relative percentage change from the null value of 1.0 using (exp(scaled log-relative effect) − 1) × 100.
4. For colorectal cancer, the exposure contrast corresponded to the highest-versus-lowest drinking-water nitrate comparison reported by Schullehner et al. [16]. For lung cancer, the exposure contrast corresponded to the 10 μg/m^3^ increase in ambient PM_2_._5_ reported by Hamra et al. [32].
5. The resulting distributions were summarized using means, medians, standard deviations, and 95% uncertainty intervals.

Thyroid cancer was evaluated separately using predefined low-, moderate-, and high-exposure scenario ranges because direct epidemiological estimates linking perchlorate exposure to thyroid cancer risk were unavailable. The ranges were author-defined hypothetical assumptions, sampled using uniform distributions solely to illustrate the effect of alternative assumed magnitudes on the simulated outputs. They were not derived from epidemiological effect estimates, and the resulting distributions do not quantify empirical uncertainty in a perchlorate–thyroid cancer association or the likelihood of increased thyroid cancer risk in Lebanon.

Simulation outputs were summarized using means, medians, standard deviations, and 95% uncertainty intervals. For colorectal and lung cancer, uncertainty reflected published epidemiological effect estimates combined with the assumed exposure-contrast scaling, whereas the thyroid analysis reflected only the predefined hypothetical scenario ranges.

### 2.6. Statistical Software

All simulations were conducted in Google Colab (Google LLC, Mountain View, CA, USA) using Python (version 3.12, Python Software Foundation, Wilmington, DE, USA). Monte Carlo simulations were implemented using NumPy (version 2.1.3) and SciPy (version 1.16.3) libraries, and data visualization was performed using Matplotlib (version 3.10.0).

The modeling framework was designed to explore plausible environmental health scenarios and the sensitivity of literature-derived cancer risk estimates to alternative exposure assumptions and should not be interpreted as establishing causal relationships between environmental exposures and cancer incidence.

The complete Python code used to generate the Monte Carlo simulations, summary statistics, threshold sensitivity analyses, and quantitative figures is provided in Supplementary Material S1 to support reproducibility of the quantitative analysis.

## 3. Results

### 3.1. Overview of the Modeling Framework

**Figure 1:**
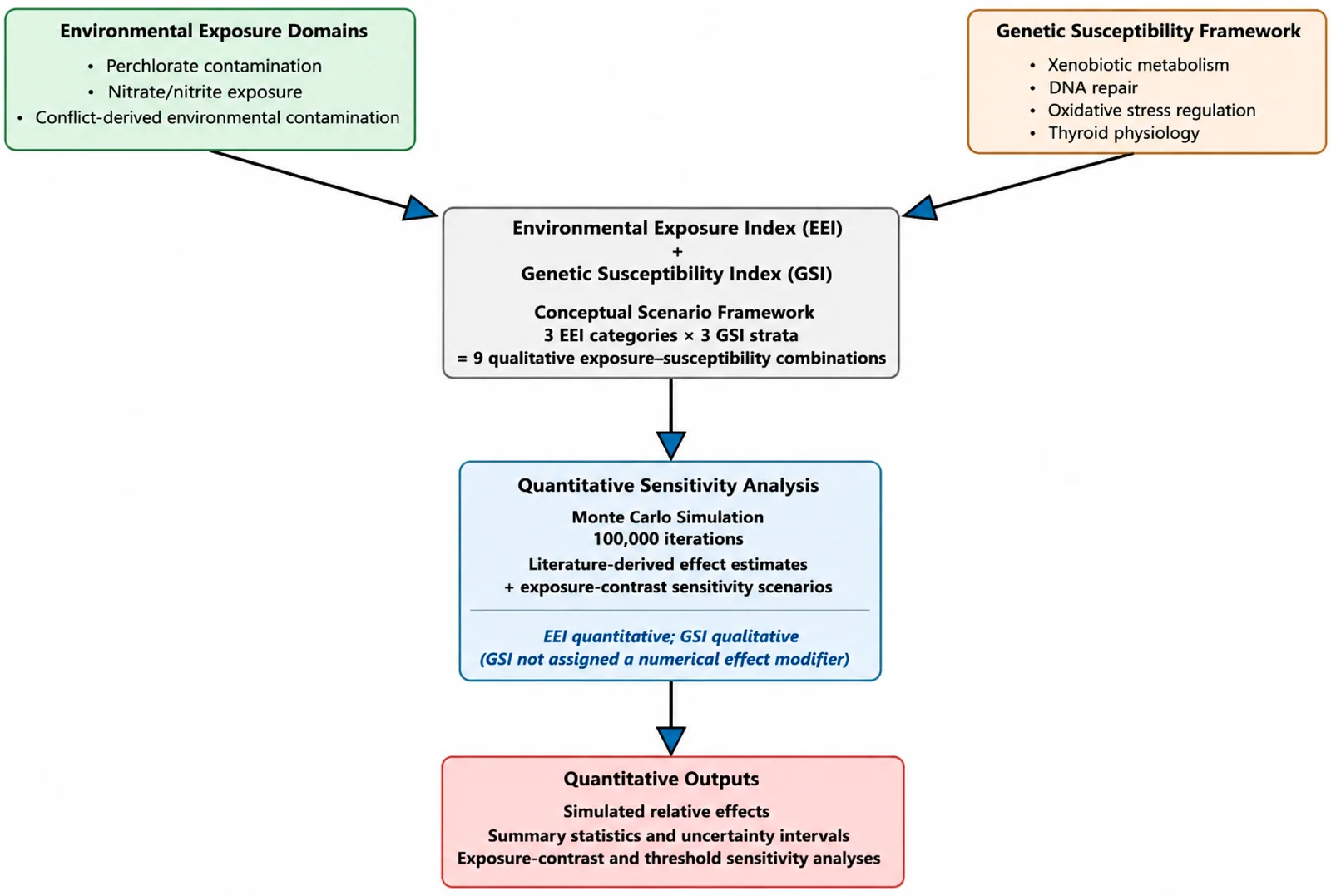
Conceptual framework linking environmental exposure scenarios and qualitative genetic susceptibility in Lebanon. Environmental exposure domains (perchlorate contamination, nitrate/nitrite exposure, and conflict-derived environmental contamination) and genetic susceptibility pathways were integrated through Environmental Exposure Index (EEI) and Genetic Susceptibility Index (GSI) classifications. Three EEI categories and three GSI strata define nine qualitative exposure–susceptibility combinations that provide the conceptual scenario structure of the framework. For the quantitative analysis, EEI categories were additionally represented by alternative exposure-contrast assumptions, whereas GSI provided a qualitative conceptual dimension and was not assigned a numerical genetic effect modifier in the Monte Carlo simulations. Monte Carlo simulations (100,000 iterations) were used to propagate uncertainty in selected literature-derived epidemiological effect estimates.

Figure 1 presents the conceptual structure of the framework, which organizes environmental exposure classifications and qualitative genetic susceptibility strata within a shared scenario structure. Detailed descriptions of the EEI and GSI classifications, model assumptions, and thyroid cancer scenario definitions are provided in Supplementary Table S1. The simulation code and analytical workflow used to generate the quantitative simulation results are provided in Supplementary Material S1.

### 3.2. Monte Carlo Simulation Outputs

**Figure 2:**
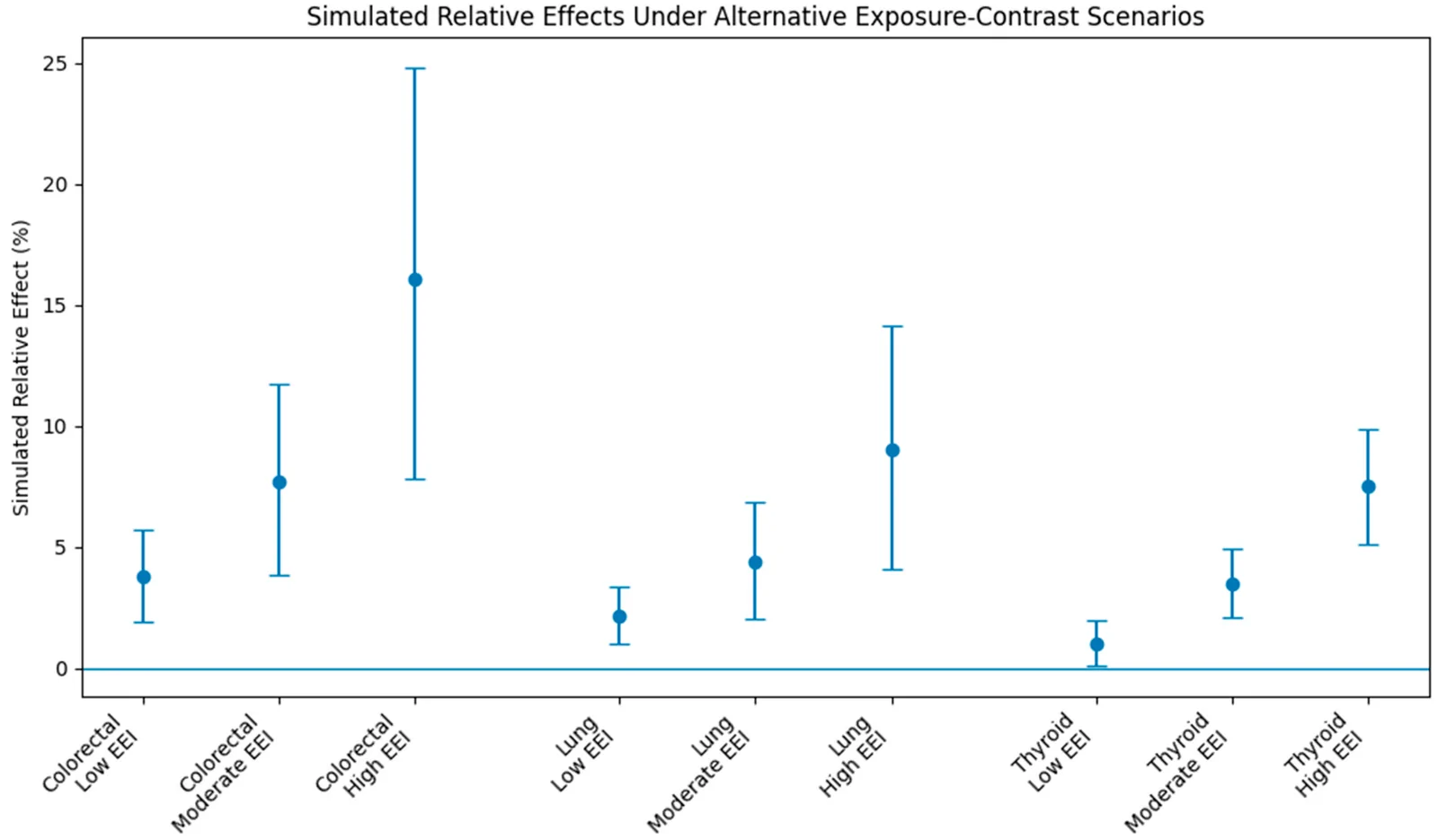
Mean simulated relative effects and corresponding 95% uncertainty intervals across low-, moderate-, and high-exposure-contrast scenarios for colorectal, lung, and thyroid cancer. Colorectal and lung cancer estimates reflect uncertainty propagated from literature-derived epidemiological effect estimates under alternative exposure-contrast assumptions, whereas thyroid cancer results represent an illustrative hypothetical sensitivity analysis based on author-defined effect ranges and are not epidemiologically parameterized risk estimates.

Monte Carlo simulations were conducted using 100,000 iterations for each cancer endpoint and exposure-contrast scenario. For colorectal and lung cancer, the quantitative analysis evaluated the sensitivity of simulated relative effects to hypothetical scaling factors of 25%, 50%, and 100% applied to the published log-relative effects; the interpretation of these factors differed by endpoint, as described in the Methods. For thyroid cancer, simulations represented the predefined assumption-based low-, moderate-, and high-exposure scenario ranges described in the Methods. Summary statistics are presented in Table 4 and visualized in Figure 2.

For colorectal cancer, the mean simulated relative effect increased progressively with the assumed exposure contrast, from 3.79% (95% UI: 1.90–5.70%) under the low-exposure-contrast scenario to 7.72% (95% UI: 3.83–11.71%) under the moderate scenario and 16.09% (95% UI: 7.81–24.80%) under the full published exposure contrast. The corresponding median values were 3.78%, 7.71%, and 16.01%, respectively. These results represent uncertainty propagated from the published colorectal cancer hazard ratio under alternative assumptions regarding the magnitude of exposure contrast and do not constitute Lebanon-specific estimates of colorectal cancer incidence or burden.

For lung cancer, the mean simulated relative effect similarly increased from 2.18% (95% UI: 1.01–3.36%) under the low-exposure-contrast scenario to 4.41% (95% UI: 2.02–6.83%) under the moderate scenario and 9.03% (95% UI: 4.09–14.13%) under the full published exposure contrast. Median values were 2.18%, 4.40%, and 9.00%, respectively. These results reflect uncertainty propagated from the published PM_2.5_–lung cancer association under alternative exposure-contrast assumptions and do not represent Lebanon-specific estimates of lung cancer incidence or burden.

Because direct epidemiological estimates linking perchlorate exposure to thyroid cancer incidence were unavailable, thyroid cancer was evaluated separately using an illustrative hypothetical sensitivity analysis. Mean simulated relative effects were 1.03% (95% UI: 0.10–1.95%), 3.50% (95% UI: 2.07–4.92%), and 7.51% (95% UI: 5.13–9.88%) under the low-, moderate-, and high-exposure scenarios, respectively. These results are conditional on the author-defined hypothetical uniform distributions and their prespecified bounds. They should not be interpreted as epidemiological estimates of perchlorate-associated thyroid cancer risk, evidence of an exposure–response relationship, or predictions of thyroid cancer risk in Lebanon.

**Figure 3:**
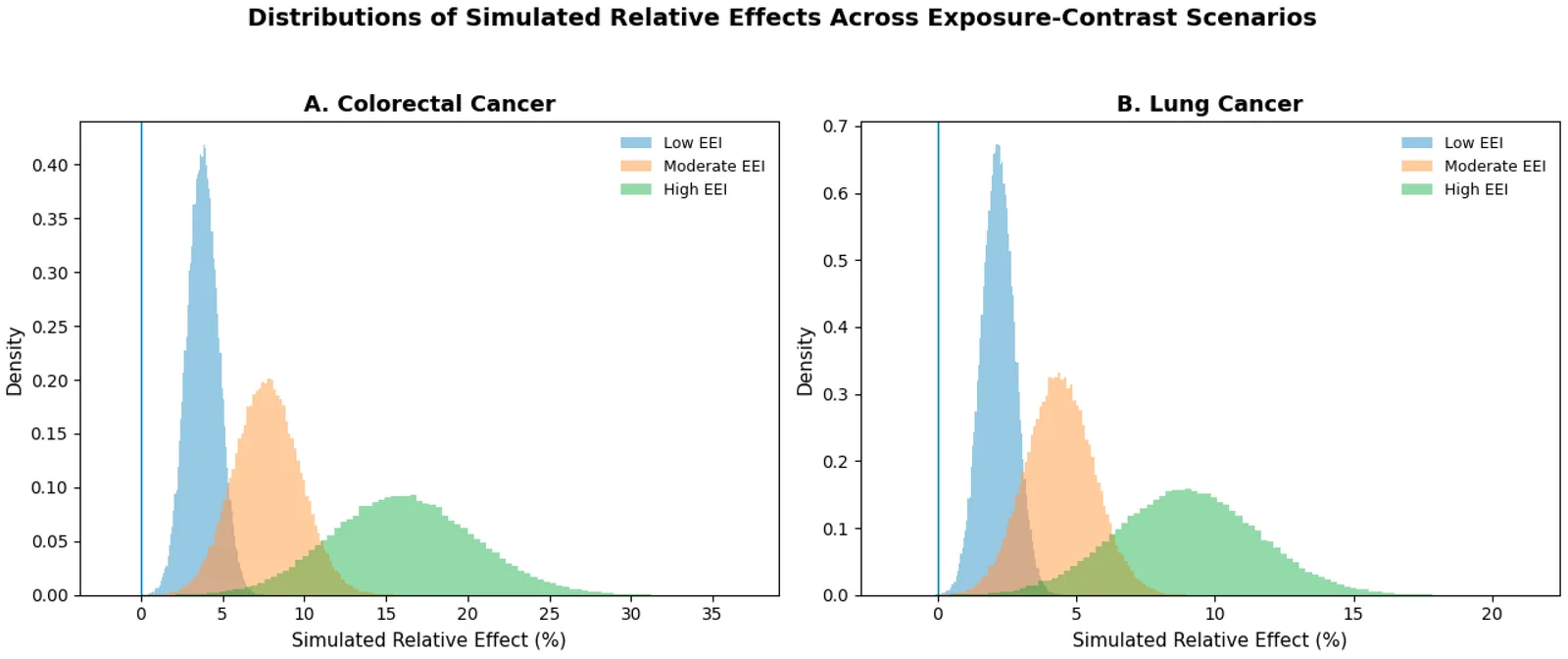
Distributions of simulated relative effects across exposure-contrast scenarios based on 100,000 Monte Carlo simulation iterations. (**A**) Colorectal cancer distributions under low-, moderate-, and high-exposure-contrast scenarios based on the hazard ratio reported by Schullehner et al. (**B**) Lung cancer distributions under low-, moderate-, and high-exposure-contrast scenarios based on the relative risk estimate reported by Hamra et al.

The distributions of simulated colorectal and lung cancer relative effects shifted progressively toward larger values as the assumed exposure contrast increased from the low- to the high-exposure-contrast scenario. The thyroid cancer distributions reflected the predefined hypothetical low-, moderate-, and high-exposure scenario assumptions. The resulting distributions are presented in Figure 3.

### 3.3. Threshold Sensitivity Analysis

For colorectal cancer, under the high-exposure-contrast scenario, 92.34% of simulated relative effects exceeded a 10% threshold, 59.20% exceeded 15%, and 18.27% exceeded 20%. Under the moderate-exposure-contrast scenario, 12.97% of simulations exceeded 10%, while 91.40% exceeded 5%. These findings reflect the distribution of uncertainty in the literature-derived colorectal cancer effect estimate under the specified exposure-contrast assumptions.

For lung cancer, under the high-exposure-contrast scenario, 94.52% of simulated relative effects exceeded a 5% threshold and 34.84% exceeded 10%. Under the moderate-exposure-contrast scenario, 31.36% exceeded 5%. These findings similarly reflect the distribution of uncertainty in the literature-derived lung cancer effect estimate under alternative exposure-contrast assumptions.

For thyroid cancer, 66.66% of simulations under the moderate-exposure scenario exceeded a 3% relative effect, while 100% of simulations under the high-exposure scenario exceeded a 5% relative effect. These proportions are conditional on the author-defined hypothetical uniform distributions and their selected bounds. They describe the fraction of simulated values exceeding predefined thresholds under those assumptions, not the probability of thyroid cancer incidence or burden increasing by the stated percentages in Lebanon.

## 4. Discussion

This study presents a scenario-based environmental cancer risk modeling framework designed to explore environmental cancer risk under conditions of exposure uncertainty in Lebanon, while considering heterogeneous biological susceptibility as a qualitative conceptual dimension. By organizing environmental exposure domains (perchlorate contamination, nitrate/nitrite exposure, and conflict-derived environmental contamination) alongside genetic susceptibility classifications, the model provides a structured approach for exploring plausible variations in environmental risk under alternative environmental scenarios. Because direct population-level environmental measurements for these contaminants were limited, the exposure domains were represented through qualitative scenarios, while the quantitative analysis evaluated alternative exposure-contrast assumptions for selected cancer endpoints using literature-derived relative effect estimates. Importantly, the results should be interpreted as an exploratory, assumption-based characterization of uncertainty around these published estimates, rather than as causal estimates of cancer risk, Lebanon-specific risk estimates, or projections of future cancer burden. The quantitative simulations did not incorporate Lebanese-specific exposure distributions or quantitatively model genetic susceptibility.

The Monte Carlo simulations demonstrated uncertainty distributions around the literature-derived relative effect estimates for colorectal and lung cancer. For colorectal cancer, the mean simulated relative effect increased from 3.79% under the low-exposure-contrast scenario to 7.72% under the moderate scenario and 16.09% under the full published exposure contrast, illustrating the sensitivity of the simulated effect distribution to the assumed magnitude of exposure. Similarly, lung cancer simulations showed mean relative effects of 2.18%, 4.41%, and 9.03% under the low-, moderate-, and high-exposure-contrast scenarios, respectively. These results illustrate how the simulated effect distributions change under the specified scaling assumptions; they do not provide independent evidence of an exposure–response relationship or estimates of cancer risk in Lebanon [32].

The threshold analysis further characterized the distributions of simulated relative effects under the specified scaling assumptions; it did not provide independent evidence of an exposure–response relationship. Under the high-exposure-contrast scenario, 92.34% of colorectal simulations exceeded a 10% relative-effect threshold, while 59.20% exceeded 15% and 18.27% exceeded 20%. For lung cancer, 94.52% of simulations under the high-exposure-contrast scenario exceeded a 5% threshold, while 34.84% exceeded 10%. These proportions describe the fraction of simulated literature-derived effect values exceeding predefined thresholds under the model assumptions. They are not probabilities that cancer incidence or burden will increase by these percentages in Lebanon. The results should therefore be interpreted as a characterization of uncertainty under hypothetical scaling assumptions, rather than as predictions of absolute future cancer incidence or independent estimates of cancer risk in the Lebanese population.

The thyroid cancer analysis was included as an illustrative hypothetical sensitivity analysis to examine how prespecified relative-effect assumptions influence model outputs across low-, moderate-, and high-exposure scenarios. Perchlorate can inhibit the sodium–iodide symporter and disrupt thyroid hormone synthesis, providing a biologically plausible basis for investigating thyroid-related effects [33,34]. However, this established mechanism of thyroid hormone disruption does not establish a quantitative effect on thyroid cancer incidence or justify the specific numerical ranges used in this analysis. Because robust epidemiological estimates linking perchlorate exposure to thyroid cancer incidence were unavailable to parameterize the model, the selected ranges were author-defined assumptions rather than literature-derived risk estimates. Consequently, the thyroid outputs should be interpreted solely as illustrations of the model’s behavior under the specified assumptions, not as estimates of thyroid cancer risk, evidence of a causal association, or projections of cancer burden in Lebanon.

A conceptual contribution of this study is the organization of environmental exposure scenarios alongside a qualitative genetic susceptibility framework. The GSI was designed to represent variability in biological responsiveness across pathways involved in xenobiotic metabolism, oxidative stress regulation, DNA repair, and thyroid physiology. Epigenetic mechanisms, including DNA methylation, histone modifications, and non-coding RNA regulation, may also influence toxicant-responsive signaling pathways and contribute to environmentally mediated susceptibility; however, these mechanisms were beyond the scope of the present genetic susceptibility framework. Importantly, the GSI was used only as a qualitative conceptual framework; it was not used to stratify the Monte Carlo simulations or to modify the quantitative effect estimates. This approach aligns with broader environmental epidemiology frameworks that recognize the importance of biological susceptibility in modifying responses to environmental exposures [2,3]. In the absence of population-specific genotype prevalence data and validated interaction coefficients for Lebanon, GSI was used to represent biologically plausible heterogeneity rather than measurable genetic risk modification.

The EEI was constructed as a scenario-based representation of environmental conditions rather than a direct measurement of exposure. This is particularly relevant in Lebanon, where systematic environmental monitoring data remain limited for groundwater contamination, air quality, and conflict-related pollutants. Accordingly, EEI categories (low, moderate, high exposure) should be interpreted as comparative environmental states rather than quantified exposure levels. The low-exposure scenario represents a hypothetical environmental state characterized by greater monitoring, remediation, regulatory control, and exposure reduction; the moderate scenario represents an intermediate hypothetical environmental condition; and the high-exposure scenario represents persistent or increasing contamination with limited mitigation. For the quantitative colorectal and lung cancer analyses, these EEI categories represented by hypothetical scaling factors of 25%, 50%, and 100% applied to the published log-relative effects. For lung cancer, these factors corresponded to hypothetical PM_2.5_ exposure contrasts of 2.5, 5, and 10 μg/m^3^ PM_2.5_ under the assumed log-linear exposure–response relationship. For colorectal cancer, the factors were applied to the published log-hazard ratio and did not represent fractions of the highest-versus-lowest drinking-water nitrate exposure contrast rather or measured Lebanese exposure levels. These categories therefore do not represent quantitative annual exposure-reduction rates or concentration trajectories, and should not be interpreted as quantitative exposure forecasts. This approach is consistent with global scenario-based environmental health modeling frameworks used in environmental health scenario modeling studies when empirical monitoring data are incomplete or unavailable [23].

Conflict-related environmental contamination represents an important but often under-characterized environmental exposure domain. Recent evidence indicates that armed conflict can generate persistent environmental contamination through multiple pathways, including particulate emissions from explosions, combustion by-products, heavy metal deposition, and destruction of industrial infrastructure [36].

Contemporary reviews further highlight that such exposures may persist long after active conflict periods, contributing to long-term soil contamination, ecosystem disruption, and degradation of environmental quality [37,38]. Within this context, inclusion of conflict-derived contamination in the present framework reflects an attempt to capture a plausible and environmentally relevant exposure domain for Lebanon, given its documented exposure to repeated armed conflict and infrastructure damage. Within this broader conceptual exposure domain, PM_2.5_ represents the component that was quantitatively parameterized in the lung cancer analysis; the published PM_2.5_ effect estimate was not used as a quantitative estimate of the cancer effect of white phosphorus or other individual conflict-related contaminants.

Several limitations should be acknowledged. First, the model does not incorporate individual-level exposure measurements or longitudinal epidemiological data from Lebanon. Instead, it relies on scenario-based constructs (EEI and GSI), which were not empirically validated. Second, the genetic susceptibility framework does not include quantitative exposure–susceptibility interaction coefficients or population-specific allele frequencies. It therefore represents a conceptual stratification tool rather than a predictive genetic risk model. Third, the thyroid cancer analysis uses author-defined hypothetical effect ranges rather than epidemiologically derived estimates. Although the analysis illustrates how these assumptions influence the simulated outputs, the selected ranges are not empirically validated and should not be interpreted as plausible bounds on the actual thyroid cancer effect of perchlorate exposure. The thyroid results are therefore particularly sensitive to the prespecified assumptions and do not provide independent evidence of thyroid cancer risk. Fourth, the quantitative simulations propagate uncertainty from the published effect estimates and the specified scaling assumptions but do not quantify other important sources of uncertainty, including differences between the source-study populations and the Lebanese population, exposure measurement error, residual confounding, between-study heterogeneity, and uncertainty in the applicability of the published associations to the Lebanese context. The hypothetical scaling assumptions introduce additional uncertainty, particularly for colorectal cancer, where the source estimate compares categories of drinking-water nitrate exposure rather than a per-unit concentration contrast. These sources of uncertainty were not independently quantified in the present analysis. In addition, the colorectal and lung cancer simulations do not incorporate Lebanese-specific exposure distributions, exposure trajectories, population projections, or latency functions and therefore should not be interpreted as estimates of future national cancer incidence or absolute cancer burden. Finally, the results should not be interpreted as causal estimates of environmental exposure effects on cancer incidence but rather as exploratory scenario-based analyses intended to characterize uncertainty, generate testable hypotheses, and inform future research and environmental health planning.

Despite these limitations, the framework has potential relevance for environmental health planning in Lebanon and similar settings characterized by environmental data scarcity and complex exposure profiles. Rather than serving as a substitute for empirical epidemiological assessment, the framework can be used to help identify exposure domains and cancer endpoints that may warrant targeted environmental monitoring, biomonitoring, epidemiological investigation, or surveillance. Strengthening environmental monitoring systems for groundwater contamination, air pollution, and post-conflict environmental assessment may improve the empirical basis for future risk modeling efforts.

Integration of environmental surveillance with cancer registry systems could support earlier identification of potential exposure-related health burdens. More broadly, the scenario-based framework presented here may assist in prioritizing environmental health research, supporting environmental surveillance strategies, and informing resource allocation in settings where direct exposure measurement is limited. For example, alternative environmental scenarios can be used to identify which exposure pathways require better characterization, where biomonitoring may be most informative, and which cancer outcomes could be prioritized for further epidemiological investigation. Such applications should be viewed as hypothesis-generating and planning tools rather than evidence of attributable cancer burden.

## 5. Conclusions

This study developed a transparent, scenario-based environmental risk modeling framework examining environmental exposure uncertainty alongside qualitative genetic susceptibility concepts to explore environmental cancer risk in Lebanon under alternative exposure conditions. By combining multiple exposure domains, including perchlorate contamination, nitrate/nitrite exposure, and conflict-derived environmental contamination, with Monte Carlo simulation methods, the framework provides a structured approach for characterizing uncertainty in selected literature-derived cancer risk estimates while incorporating qualitative environmental and susceptibility scenarios. Given its scenario-based structure, the framework may also be adapted to other countries or settings characterized by similar environmental exposure profiles, data limitations, and heterogeneous susceptibility, with exposure domains and model parameters tailored to the local context.

The model is not intended to establish causal relationships or provide predictions of future cancer incidence; rather, it offers a probabilistic approach for characterizing plausible environmental health scenarios and identifying potential areas of concern. The findings demonstrate that alternative assumptions regarding exposure contrast can substantially influence the distribution of simulated literature-derived relative effects, while the genetic susceptibility component provides a qualitative framework for considering biological heterogeneity. These findings should not be interpreted as estimates of cancer incidence, population-attributable risk, or causal environmental effects in Lebanon. The thyroid cancer component was an illustrative hypothetical sensitivity analysis based on author-defined assumptions, rather than a quantitative estimate of perchlorate-associated thyroid cancer risk. Its results should therefore be interpreted as demonstrations of model behavior under specified assumptions, not as evidence of future thyroid cancer risk in Lebanon.

The practical value of the framework lies in its potential to support environmental health planning and surveillance prioritization rather than to estimate attributable cancer burden. It may help identify priority exposure pathways for environmental monitoring, inform the design of biomonitoring and epidemiological studies, and guide hypotheses regarding cancer outcomes that warrant further investigation. In this context, the framework complements rather than replaces empirical exposure assessment and longitudinal epidemiological research.

Future research should prioritize the integration of empirical environmental monitoring data, population-specific genetic information, longitudinal epidemiological studies, and validated exposure–response relationships to improve model precision and strengthen causal inference. Future iterations could also incorporate quantitative exposure trajectories, population projections, validated gene–environment interaction parameters, and cancer-specific latency assumptions once these data become available. As environmental health data systems continue to develop, this framework may serve as a foundation for more refined environmental cancer risk assessments in Lebanon and other data-limited settings.

## Figures and Tables

**Table 1:** Candidate Genes Representing Exposure-Relevant Biological Pathways in the Qualitative Genetic Susceptibility Framework.

Gene	Biological Function	Relevant Cancer Pathway/Endpoint	Reference
GSTM1 (Glutathione S-transferase Mu 1)	Glutathione-dependent conjugation and detoxification of electrophilic xenobiotics	Colorectal, Lung	[26]
GSTT1 (Glutathione S-transferase Theta 1)	Glutathione-dependent conjugation and detoxification of electrophilic xenobiotics	Colorectal	[26]
NAT2 (N-acetyltransferase 2)	Phase II acetylation of aromatic amines and related xenobiotic compounds	Colorectal	[27]
XRCC1 (X-ray repair cross-complementing protein 1)	Scaffold protein involved in base excision repair and single-strand DNA break repair	Lung	[28]
CYP1A1 (Cytochrome P450 Family 1 Subfamily A Member 1)	Phase I oxidative metabolism and bioactivation of polycyclic aromatic hydrocarbons and other procarcinogens	Lung	[29]
SLC5A5 (Solute Carrier Family 5 Member 5; Sodium/Iodide Symporter)	Sodium–iodide symporter mediating active iodide uptake into thyroid follicular cells	Thyroid	[30,31]

**Table 2:** Qualitative Environmental Exposure–Genetic Susceptibility Scenario Matrix.

Environmental Exposure Index (EEI)	Low Susceptibility	Intermediate Susceptibility	High Susceptibility
Low exposure (mitigation)	Low EEI × Low GSI	Low EEI × Intermediate GSI	Low EEI × High GSI
Moderate exposure (intermediate hypothetical scenario)	Moderate EEI × Low GSI	Moderate EEI × Intermediate GSI	Moderate EEI × High GSI
High exposure (persistent contamination)	High EEI × Low GSI	High EEI × Intermediate GSI	High EEI × High GSI

**Table 3:** Epidemiological Effect Estimates and Hypothetical Assumptions Used in the Quantitative Sensitivity Analyses.

Exposure Domain	Cancer Endpoint	Source	Exposure Contrast	Effect Estimate (95% CI)	Distribution
Nitrate/Nitrite Exposure	Colorectal Cancer	Schullehner et al., 2018 [16]	Highest vs. lowest drinking-water nitrate exposure	HR 1.16 (1.08–1.25)	Log-normal
Conflict-Derived Environmental Contamination (PM_2.5_ component)	Lung Cancer	Hamra et al., 2014 [32]	Per 10 μg/m^3^ increase in ambient PM_2.5_	RR 1.09 (1.04–1.14)	Log-normal
Perchlorate Exposure	Thyroid Cancer	Leung et al., 2010 [33]; Steinmaus, 2016 [34]; Niziński et al., 2021 [35]	No direct epidemiological estimate	Author-defined hypothetical ranges: 0.05–2%, 2–5%, and 5–10%	Uniform within predefined scenario range

**Table 4:** Summary Statistics of Simulated Relative Effects Across Exposure-Contrast Scenarios.

Cancer Endpoint	EEI Scenario	Mean (%)	Median (%)	SD	95% UI (%)
Colorectal	Low	3.79	3.78	0.97	1.90–5.70
Colorectal	Moderate	7.72	7.71	2.01	3.83–11.71
Colorectal	High	16.09	16.01	4.33	7.81–24.80
Lung	Low	2.18	2.18	0.60	1.01–3.36
Lung	Moderate	4.41	4.40	1.22	2.02–6.83
Lung	High	9.03	9.00	2.55	4.09–14.13
Thyroid	Low	1.03	1.02	0.56	0.10–1.95
Thyroid	Moderate	3.50	3.50	0.87	2.07–4.92
Thyroid	High	7.51	7.51	1.44	5.13–9.88

## Data Availability

The original contributions presented in this study are included in the article/Supplementary Material. Further inquiries can be directed to the corresponding authors.

## Supplementary Materials

The following supporting information can be downloaded at https://www.mdpi.com/article/10.3390/toxics14100921/s1, Supplementary Table S1: Model Assumptions and Scenario Definitions; Supplementary Material S1: Monte Carlo Simulation and Exposure-Contrast Sensitivity Analysis.

## Abbreviations

The following abbreviations are used in this manuscript:

GSI	Genetic susceptibility index
EEI	Environmental Exposure Index
IARC	International Agency for Research on Cancer
GSTM1	Glutathione S-transferase Mu 1
GSTT1	Glutathione S-transferase Theta 1
NAT2	N-acetyltransferase 2
XRCC1	X-ray repair cross-complementing protein 1
CYP1A1	Cytochrome P450 Family 1 Subfamily A Member 1
SLC5A5	Solute Carrier Family 5 Member 5 (Sodium/Iodide Symporter)
