# Supplementary material for: Environmental Cancer Risk Scenarios for Lebanon: Quantitative Sensitivity Analysis and a Qualitative Genetic Susceptibility Framework
Source: Toxics. 2026 Oct 17;14(10):921. doi: 10.3390/toxics14100921 (PMC13709853; doi:10.3390/toxics14100921)
Supplement: Supplementary file 1 [file toxics-14-00921-s001.zip › Supplementary Material S1.pdf]

```
# ============================================================
# SUPPLEMENTARY MATERIAL S1
# Monte Carlo Simulation and Exposure-Contrast Sensitivity Analysis
# ============================================================

import numpy as np
import pandas as pd
import matplotlib.pyplot as plt
from scipy.stats import lognorm

# ============================================================
# 1. SETTINGS
# ============================================================

np.random.seed(42)
n_sim = 100000

# ============================================================
# 2. EXPOSURE-CONTRAST SENSITIVITY SCENARIOS
# ============================================================
#
# These scenarios use hypothetical scaling factors applied
# to published log-relative effect estimates. They are NOT
# measured Lebanese exposures or exposure distributions.
#
# Low EEI      = 25% of the published log-relative effect
# Moderate EEI = 50% of the published log-relative effect
# High EEI     = 100% of the published log-relative effect
#
# For colorectal cancer (CRC), the source estimate compares
# the highest versus lowest categories of long-term
# drinking-water nitrate exposure. The scaling factors are
# applied to the log-HR; they do NOT define fractions of the
# underlying nitrate concentration contrast.
#
# For lung cancer, the source estimate is an RR per 10 µg/m³
# increase in ambient PM2.5. Under the assumed log-linear
# relationship, the scaling factors correspond mathematically
# to hypothetical contrasts of 2.5, 5, and 10 µg/m³.
#
# These are sensitivity assumptions, not Lebanon-specific
# exposure-response functions or measured exposure levels.

exposure_scenarios = {
    "Low EEI": 0.25,
    "Moderate EEI": 0.50,
    "High EEI": 1.00
}
```

```
# ============================================================
# 3. LITERATURE-DERIVED EPIDEMIOLOGICAL PARAMETERS
# ============================================================

# Colorectal cancer
# Schullehner et al., 2018
# HR = 1.16 (95% CI 1.08–1.25)
# Contrast: highest versus lowest categories of long-term
# drinking-water nitrate exposure.

crc_hr_point = 1.16
crc_hr_low = 1.08
crc_hr_high = 1.25

# Lung cancer
# Hamra et al., 2014
# RR = 1.09 (95% CI 1.04–1.14)
# Contrast: per 10 μg/m³ increase in ambient PM2.5.
#
# PM2.5 is the quantitatively parameterized component of the
# broader conflict-derived environmental contamination domain.
# This estimate is NOT an estimate of white phosphorus-related
# lung cancer risk.

lung_rr_point = 1.09
lung_rr_low = 1.04
lung_rr_high = 1.14

# ============================================================
# 4. LOGNORMAL PARAMETERIZATION
# ============================================================
#
# The published point estimate is used as the median of the
# fitted lognormal distribution. The published 95% CI is used
# to estimate the standard deviation on the logarithmic scale.
#
# These distributions approximate uncertainty in the published
# epidemiological effect estimates. They do not capture all
# sources of uncertainty, such as source-population differences,
# residual confounding, exposure measurement error, or the
# transferability of published associations to Lebanon.
# ============================================================

def get_lognormal_params(point_estimate, lower_ci, upper_ci):
    sigma = (
        np.log(upper_ci) - np.log(lower_ci)
    ) / (2 * 1.96)
```

```
    mu = np.log(point_estimate)
    return mu, sigma

# ============================================================
# 5. SAMPLE LITERATURE EFFECT DISTRIBUTIONS
# ============================================================

# Colorectal cancer
mu_crc, sigma_crc = get_lognormal_params(
    crc_hr_point,
    crc_hr_low,
    crc_hr_high
)

crc_hr_samples = lognorm(
    s=sigma_crc,
    scale=np.exp(mu_crc)
).rvs(n_sim)

# Lung cancer
mu_lung, sigma_lung = get_lognormal_params(
    lung_rr_point,
    lung_rr_low,
    lung_rr_high
)

lung_rr_samples = lognorm(
    s=sigma_lung,
    scale=np.exp(mu_lung)
).rvs(n_sim)

# ============================================================
# 6. EXPOSURE-CONTRAST SCALING
# ============================================================
#
# The logarithm of each sampled relative effect is scaled:
#
#   RR_scenario = exp[f * ln(RR_sampled)]
#
# where f = 0.25, 0.50, or 1.00.
#
# For CRC, this is a mathematical scaling of the published
# log-HR comparing the highest and lowest nitrate exposure
# categories. It does NOT define a specific nitrate
# concentration contrast.
#
# For lung cancer, the factors correspond to hypothetical
```

```
# PM2.5 contrasts of 2.5, 5, and 10 µg/m³ under the assumed
# log-linear exposure-response relationship.
#
# These are exploratory sensitivity assumptions, not a
# Lebanon-specific exposure-response function.
# ============================================================

crc_results = {}
lung_results = {}

for scenario, fraction in exposure_scenarios.items():

    # Colorectal cancer
    crc_scenario_rr = np.exp(
        fraction * np.log(crc_hr_samples)
    )

    crc_percent_change = (
        crc_scenario_rr - 1
    ) * 100

    crc_results[scenario] = crc_percent_change

    # Lung cancer
    lung_scenario_rr = np.exp(
        fraction * np.log(lung_rr_samples)
    )

    lung_percent_change = (
        lung_scenario_rr - 1
    ) * 100

    lung_results[scenario] = lung_percent_change

# ============================================================
# 7. THYROID CANCER EXPLORATORY SCENARIO ANALYSIS
# ============================================================
#
# Direct epidemiological estimates linking perchlorate
# exposure to thyroid cancer incidence were unavailable.
#
# Thyroid cancer is therefore evaluated separately using
# author-defined hypothetical relative-effect ranges:
#
# Low scenario:      0.05%–2%
# Moderate scenario: 2%–5%
# High scenario:     5%–10%
#
```

```
# Uniform distributions are used within these predefined
# ranges solely to illustrate how the assumed values affect
# the simulated outputs.
#
# These ranges are NOT derived from epidemiological effect
# estimates or validated quantitative mechanistic models.
# Mechanistic evidence on thyroid hormone disruption provides
# biological context but does not validate these numerical
# assumptions.
#
# The outputs must NOT be interpreted as estimates of
# perchlorate-associated thyroid cancer risk or predictions
# of thyroid cancer risk or burden in Lebanon.
# ============================================================

thyroid_low = np.random.uniform(
    0.0005,
    0.02,
    n_sim
)

thyroid_moderate = np.random.uniform(
    0.02,
    0.05,
    n_sim
)

thyroid_high = np.random.uniform(
    0.05,
    0.10,
    n_sim
)

thyroid_results = {
    "Low EEI": thyroid_low * 100,
    "Moderate EEI": thyroid_moderate * 100,
    "High EEI": thyroid_high * 100
}

# ============================================================
# 8. SUMMARY STATISTICS
# ============================================================

def summarize(data):
    return {
        "Mean (%)": np.mean(data),
        "Median (%)": np.median(data),
        "SD": np.std(data),
```

```
        "Lower 95% UI (%)": np.percentile(data, 2.5),
        "Upper 95% UI (%)": np.percentile(data, 97.5)
    }

summary_rows = []

# Colorectal cancer
for scenario in exposure_scenarios:
    stats = summarize(crc_results[scenario])

    summary_rows.append({
        "Cancer Endpoint": "Colorectal",
        "EEI Scenario": scenario,
        **stats
    })

# Lung cancer
for scenario in exposure_scenarios:
    stats = summarize(lung_results[scenario])

    summary_rows.append({
        "Cancer Endpoint": "Lung",
        "EEI Scenario": scenario,
        **stats
    })

# Thyroid cancer
for scenario in exposure_scenarios:
    stats = summarize(thyroid_results[scenario])

    summary_rows.append({
        "Cancer Endpoint": "Thyroid",
        "EEI Scenario": scenario,
        **stats
    })

summary_df = pd.DataFrame(summary_rows)

# ============================================================
# 9. THRESHOLD SENSITIVITY ANALYSIS
# ============================================================
#
# These values represent the percentage of simulated relative
# effects exceeding predefined numerical thresholds.
#
# They describe the simulated distributions conditional on
# the model assumptions. They are NOT probabilities of cancer
# incidence or burden increasing by these amounts in Lebanon.
```

```
# ============================================================

thresholds_crc = [5, 10, 15, 20]
thresholds_lung = [5, 10, 15]

threshold_rows = []

# CRC threshold analysis
for scenario in exposure_scenarios:
    data = crc_results[scenario]

    for threshold in thresholds_crc:
        proportion = np.mean(data > threshold) * 100

        threshold_rows.append({
            "Cancer Endpoint": "Colorectal",
            "EEI Scenario": scenario,
            "Threshold (%)": threshold,
            "Proportion Above Threshold (%)": proportion
        })

# Lung threshold analysis
for scenario in exposure_scenarios:
    data = lung_results[scenario]

    for threshold in thresholds_lung:
        proportion = np.mean(data > threshold) * 100

        threshold_rows.append({
            "Cancer Endpoint": "Lung",
            "EEI Scenario": scenario,
            "Threshold (%)": threshold,
            "Proportion Above Threshold (%)": proportion
        })

# Thyroid threshold analysis
# These proportions are conditional on the predefined
# hypothetical uniform distributions and their selected bounds.

thyroid_thresholds = {
    "Moderate EEI": [3],
    "High EEI": [5]
}

for scenario, thresholds in thyroid_thresholds.items():
    data = thyroid_results[scenario]

    for threshold in thresholds:
```

```
        proportion = np.mean(data > threshold) * 100

        threshold_rows.append({
            "Cancer Endpoint": "Thyroid",
            "EEI Scenario": scenario,
            "Threshold (%)": threshold,
            "Proportion Above Threshold (%)": proportion
        })

threshold_df = pd.DataFrame(threshold_rows)

# ============================================================
# 10. DISPLAY RESULTS
# ============================================================

print("\n")
print("=" * 80)
print("SUMMARY STATISTICS")
print("=" * 80)

print(
    summary_df.round(2).to_string(index=False)
)

print("\n")
print("=" * 80)
print("THRESHOLD SENSITIVITY ANALYSIS")
print("=" * 80)

print(
    threshold_df.round(2).to_string(index=False)
)

# ============================================================
# 11. SAVE NUMERICAL RESULTS
# ============================================================

summary_df.to_csv(
    "MonteCarlo_Scenario_Summary.csv",
    index=False
)

threshold_df.to_csv(
    "MonteCarlo_Threshold_Sensitivity.csv",
    index=False
)

# ============================================================
```

```
# 12. FIGURE 2
# ============================================================
#
# Mean simulated relative effect with percentile-based
# intervals across alternative scenarios. For CRC and lung,
# the intervals reflect propagated uncertainty from the
# published estimates under the stated assumptions. For
# thyroid cancer, they reflect only the hypothetical ranges.
# ============================================================

fig, ax = plt.subplots(figsize=(10, 6))

cancer_order = ["Colorectal", "Lung", "Thyroid"]

scenario_order = [
    "Low EEI",
    "Moderate EEI",
    "High EEI"
]

x_positions = []
means = []
lower_errors = []
upper_errors = []
labels = []

x = 0

for cancer in cancer_order:
    for scenario in scenario_order:

        row = summary_df[
            (summary_df["Cancer Endpoint"] == cancer) &
            (summary_df["EEI Scenario"] == scenario)
        ].iloc[0]

        mean = row["Mean (%)"]
        lower = row["Lower 95% UI (%)"]
        upper = row["Upper 95% UI (%)"]

        x_positions.append(x)
        means.append(mean)
        lower_errors.append(mean - lower)
        upper_errors.append(upper - mean)

        labels.append(f"{cancer}\n{scenario}")
        x += 1
```

```
    x += 0.5

ax.errorbar(
    x_positions,
    means,
    yerr=[lower_errors, upper_errors],
    fmt="o",
    capsize=5,
    linewidth=1.5
)

ax.set_xticks(x_positions)
ax.set_xticklabels(labels, rotation=45, ha="right")

ax.set_ylabel("Simulated Relative Effect (%)")
ax.set_title(
    "Simulated Relative Effects Under Alternative "
    "Exposure-Contrast Scenarios"
)

ax.axhline(0, linewidth=1)

plt.tight_layout()

plt.savefig(
    "Figure2_Exposure_Sensitivity.png",
    dpi=600,
    bbox_inches="tight"
)

plt.savefig(
    "Figure2_Exposure_Sensitivity.pdf",
    dpi=600,
    bbox_inches="tight"
)

plt.show()

# ============================================================
# 13. FIGURE 3
# ============================================================
#
# Distribution of simulated relative effects for colorectal
# and lung cancer across exposure-contrast scenarios.
# ============================================================

fig, axes = plt.subplots(1, 2, figsize=(13, 5.5))
```

```
# Panel A — Colorectal cancer
ax = axes[0]

for scenario in scenario_order:
    ax.hist(
        crc_results[scenario],
        bins=100,
        density=True,
        alpha=0.40,
        label=scenario
    )

ax.set_title(
    "A. Colorectal Cancer",
    fontsize=13,
    fontweight="bold"
)

ax.set_xlabel("Simulated Relative Effect (%)", fontsize=11)
ax.set_ylabel("Density", fontsize=11)
ax.axvline(0, linewidth=1)
ax.legend(frameon=False, fontsize=9)

# Panel B — Lung cancer
ax = axes[1]

for scenario in scenario_order:
    ax.hist(
        lung_results[scenario],
        bins=100,
        density=True,
        alpha=0.40,
        label=scenario
    )

ax.set_title(
    "B. Lung Cancer",
    fontsize=13,
    fontweight="bold"
)

ax.set_xlabel("Simulated Relative Effect (%)", fontsize=11)
ax.set_ylabel("Density", fontsize=11)
ax.axvline(0, linewidth=1)
ax.legend(frameon=False, fontsize=9)

fig.suptitle(
    "Distributions of Simulated Relative Effects Across "
```

```
    "Exposure-Contrast Scenarios",
    fontsize=14,
    fontweight="bold"
)

plt.tight_layout(rect=[0, 0, 1, 0.94])

plt.savefig(
    "Figure3_CRC_Lung_Distributions.png",
    dpi=600,
    bbox_inches="tight"
)

plt.savefig(
    "Figure3_CRC_Lung_Distributions.pdf",
    dpi=600,
    bbox_inches="tight"
)

plt.show()

# ============================================================
# 14. FINAL OUTPUT FILES
# ============================================================

print("\n")
print("=" * 80)
print("FILES GENERATED")
print("=" * 80)

print("1. MonteCarlo_Scenario_Summary.csv")
print("2. MonteCarlo_Threshold_Sensitivity.csv")
print("3. Figure2_Exposure_Sensitivity.png")
print("4. Figure2_Exposure_Sensitivity.pdf")
print("5. Figure3_CRC_Lung_Distributions.png")
print("6. Figure3_CRC_Lung_Distributions.pdf")

print("\nSimulation completed successfully.")
```
