# Supplementary material for: Environmental Cancer Risk Scenarios for Lebanon: Quantitative Sensitivity Analysis and a Qualitative Genetic Susceptibility Framework
Source: Toxics. 2026 Oct 17;14(10):921. doi: 10.3390/toxics14100921 (PMC13709853; doi:10.3390/toxics14100921)
Supplement: Supplementary file 1 [file toxics-14-00921-s001.zip › Supplementary Table S1.pdf]

Supplementary Table S1. Model Assumptions and Scenario Definitions

Component	Category	Definition / Assumption	Source or Justification
Environmental Exposure Index (EEI)	Low Exposure Scenario	Represents a hypothetical environmental condition associated conceptually with greater monitoring, remediation, regulatory control, and exposure reduction. In the quantitative colorectal and lung cancer sensitivity analyses, the low scenario applies a scaling factor of 25% to the published log-relative effect estimate.	Scenario-based sensitivity assumption
EEI	Moderate Exposure Scenario	Represents an intermediate hypothetical environmental condition and does not imply that this scenario reflects current measured conditions in Lebanon. In the quantitative colorectal and lung cancer sensitivity analyses, the moderate scenario applies a scaling factor of 50% to the published log-relative effect estimate.	Scenario-based sensitivity assumption
EEI	High Exposure Scenario	Represents a hypothetical environmental condition characterized conceptually by persistent contamination and limited mitigation. In the quantitative colorectal and lung cancer sensitivity analyses, the high scenario applies a scaling factor of 100% to the published log-relative effect estimate.	Scenario-based sensitivity assumption
EEI Quantitative Scaling	Exposure-Contrast Sensitivity Analysis	The 25%, 50%, and 100% values are hypothetical scaling factors applied to the logarithm of the published relative effect estimate. For colorectal cancer, the factors scale the published log-HR comparing the highest and lowest categories of drinking-water nitrate exposure; they do not define fractions of the underlying nitrate concentration contrast. For lung cancer, the factors correspond to hypothetical $PM_{2.5}$ contrasts of 2.5, 5, and 10 μg/m³ under	Exploratory sensitivity-analysis assumption

Component	Category	Definition / Assumption	Source or Justification
		the assumed log-linear exposure–response relationship. None of these assumptions represents a measured Lebanese exposure distribution.	
Genetic Susceptibility Index (GSI)	Low Susceptibility Profile	Represents a qualitative conceptual susceptibility profile characterized by comparatively lower biological susceptibility to environmentally mediated carcinogenesis. It is not a measured individual or population-level genetic profile.	Gene–environment interaction and exposome frameworks [1,2]
GSI	Intermediate Susceptibility Profile	Represents a qualitative conceptual profile corresponding to intermediate or average susceptibility. It is not based on measured Lebanese genotype frequencies or quantitative genetic effect estimates.	Gene–environment interaction and exposome frameworks [1,2]
GSI	High Susceptibility Profile	Represents a qualitative conceptual susceptibility profile characterized by comparatively greater susceptibility across pathways potentially involved in xenobiotic metabolism, oxidative stress regulation, DNA repair, and thyroid physiology. It does not represent a measured or validated genetic classification.	Gene–environment interaction and exposome frameworks [1,2]
GSI Classification	Qualitative Framework	GSI categories represent qualitative susceptibility strata and are not assigned genotype frequencies, numerical genetic effect modifiers, or quantitative gene–environment interaction coefficients. The GSI is not a numerical input to the Monte Carlo simulations and does not modify the simulated relative effects.	Exploratory qualitative framework
Thyroid Cancer Exploratory Scenario Parameter	Low Scenario	Author-defined hypothetical relative-effect range of 0.05–2%, sampled using a uniform distribution.	Illustrative sensitivity-analysis assumption; not derived from

Component	Category	Definition / Assumption	Source or Justification
			epidemiological effect estimates
Thyroid Cancer Exploratory Scenario Parameter	Moderate Scenario	Author-defined hypothetical relative-effect range of 2–5%, sampled using a uniform distribution.	Illustrative sensitivity-analysis assumption; not derived from epidemiological effect estimates
Thyroid Cancer Exploratory Scenario Parameter	High Scenario	Author-defined hypothetical relative-effect range of 5–10%, sampled using a uniform distribution.	Illustrative sensitivity-analysis assumption; not derived from epidemiological effect estimates
Colorectal Cancer Risk Parameter	Nitrate/Nitrite Exposure	Hazard ratio parameterized using a published epidemiological estimate. The source estimate compares the highest versus lowest categories of long-term drinking-water nitrate exposure.	Schullehner et al., 2018 [3]
Colorectal Cancer Exposure Scaling	Low / Moderate / High	Scaling factors of 25%, 50%, and 100%, respectively, applied to the logarithm of the published HR. This is a hypothetical mathematical scaling assumption and does not define corresponding nitrate concentration contrasts.	Scenario-based sensitivity analysis using Schullehner et al., 2018 [3]
Lung Cancer Risk Parameter	Conflict-Derived Environmental Contamination ($PM_{2.5}$ Component)	Relative risk parameterized using a published epidemiological estimate for ambient $PM_{2.5}$ exposure. This parameter does not estimate the effect of white phosphorus or another specific conflict-related contaminant.	Hamra et al., 2014 [4]
Lung Cancer Exposure Scaling	Low / Moderate / High	Scaling factors of 25%, 50%, and 100%, respectively, applied to the logarithm of the published RR per 10 μg/m³ increase in ambient $PM_{2.5}$. Under the assumed log-linear exposure–response relationship, these factors correspond mathematically to hypothetical contrasts	Scenario-based sensitivity analysis using Hamra et al., 2014 [4]

Component	Category	Definition / Assumption	Source or Justification
		of 2.5, 5, and 10 μg/m³. These are not measured Lebanese exposure levels.	
Monte Carlo Simulation	Iterations	100,000 iterations for each cancer endpoint and scenario.	Modeling design
Monte Carlo Simulation	Distribution	Lognormal distributions were used for the colorectal cancer HR and lung cancer RR parameters, based on published point estimates and corresponding 95% confidence intervals. The thyroid cancer analysis used separate uniform distributions within author-defined hypothetical scenario ranges.	Probabilistic uncertainty propagation for colorectal and lung cancer; illustrative hypothetical sensitivity analysis for thyroid cancer
Monte Carlo Simulation	Outcome Metric	Scenario-specific relative effect expressed as percentage change from the null value of 1.0, calculated as (scenario-specific relative effect − 1) × 100. The relative effect is an HR for colorectal cancer and an RR for lung cancer.	Analytical definition
Thyroid Cancer Analysis	Distribution	Uniform distributions were used within the predefined low-, moderate-, and high-scenario ranges. The resulting percentiles describe the selected hypothetical ranges rather than empirically estimated uncertainty in a perchlorate–thyroid cancer association.	Illustrative hypothetical sensitivity analysis
Overall Interpretation	Model Scope	The quantitative analyses characterize uncertainty and sensitivity in published relative effects for colorectal and lung cancer and explore hypothetical assumptions for thyroid cancer. The model does not estimate absolute cancer incidence, population-attributable risk, future Lebanese cancer burden, or causal effects of environmental exposures in Lebanon. The GSI is qualitative and is not quantitatively incorporated into the simulations.	Analytical scope and limitations

Note: The thyroid cancer ranges are author-defined hypothetical assumptions and are not derived from epidemiological effect estimates. Mechanistic evidence concerning perchlorate-related thyroid hormone disruption provides biological context but does not validate the selected numerical ranges. The thyroid outputs should therefore not be interpreted as estimates of perchlorate-associated thyroid cancer risk or predictions of thyroid cancer risk in Lebanon.

References

1. Thomas, D. Gene–environment-wide association studies: emerging approaches. *Nature Reviews Genetics* **2010**, *11*, 259–272.
2. Wild, C. P. The exposome: from concept to utility. *International Journal of Epidemiology* **2012**, *41*, 24–32.
3. Schullehner, J.; Hansen, B.; Thygesen, M.; Pedersen, C. B.; Sigsgaard, T. Nitrate in drinking water and colorectal cancer risk: A nationwide population-based cohort study. *International Journal of Cancer* **2018**, *143*, 73–79. https://doi.org/10.1002/ijc.31306.
4. Hamra, G. B.; Guha, N.; Cohen, A.; Laden, F.; Raaschou-Nielsen, O.; Samet, J. M.; Vineis, P.; Forastiere, F.; Saldiva, P.; Yorifuji, T. Outdoor particulate matter exposure and lung cancer: A systematic review and meta-analysis. *Environmental Health Perspectives* **2014**, *122*, 906–911.
